# Milk Source Identification and Milk Quality Estimation Using an Electronic Nose and Machine Learning Techniques

**DOI:** 10.3390/s20154238

**Published:** 2020-07-30

**Authors:** Fanglin Mu, Yu Gu, Jie Zhang, Lei Zhang

**Affiliations:** 1 School of Artificial Intelligence, Hebei University of Technology, Tianjin 300130, China; mufanglin111@163.com (F.M.); guyu@mail.buct.edu.cn (Y.G.); 2 School of Engineering, Merz Court, Newcastle University, Newcastle upon Tyne NE1 7RU, UK; jie.zhang@newcastle.ac.uk

**Keywords:** electronic nose, milk, quality estimation, source identification

## Abstract

In this study, an electronic nose (E-nose) consisting of seven metal oxide semiconductor sensors is developed to identify milk sources (dairy farms) and to estimate the content of milk fat and protein which are the indicators of milk quality. The developed E-nose is a low cost and non-destructive device. For milk source identification, the features based on milk odor features from E-nose, composition features (Dairy Herd Improvement, DHI analytical data) from DHI analysis and fusion features are analyzed by principal component analysis (PCA) and linear discriminant analysis (LDA) for dimension reduction and then three machine learning algorithms, logistic regression (LR), support vector machine (SVM), and random forest (RF), are used to construct the classification model of milk source (dairy farm) identification. The results show that the SVM model based on the fusion features after LDA has the best performance with the accuracy of 95%. Estimation model of the content of milk fat and protein from E-nose features using gradient boosting decision tree (GBDT), extreme gradient boosting (XGBoost), and random forest (RF) are constructed. The results show that the RF models give the best performance (R^2^ = 0.9399 for milk fat; R^2^ = 0.9301 for milk protein) and indicate that the proposed method in this study can improve the estimation accuracy of milk fat and protein, which provides a technical basis for predicting the quality of milk.

## 1. Introduction

Milk contains more than 100 chemical ingredients such as water, fat, phospholipids, proteins, lactose, inorganic salts, and other primary compounds [1,2]. The composition of milk is very complex. The mixture of lower fatty acids, acetones, acetaldehydes, carbon dioxide, and other volatile substances affects the odor of milk. Among them, sulfide is the main component of fresh milk odor. The flavor substances in milk are influenced by many factors, mainly produced by four forms, one of which is the reaction of milk fat, milk protein, and carbonic acid, etc. Triacylglycerols, fatty acids, diacylglycerides, saturated/polyunsaturated, and phospholipids in milk fat are directly related to the flavor of milk [3,4]. The degradation products of protein, fat, and lactose in milk are fatty acids, sulfur-containing amino acids, thiamine, etc. The decomposition process of these substances will produce volatile compounds [5,6,7]. Due to the different feed and growth environment of the cows from each dairy farm, the odor of milk produced is quite different [8]. The content of milk protein and milk fat plays a significant role in milk quality evaluation. The process of degradation for milk fat and milk protein or the interaction between derivatives can affect the milk’s odor compounds [9]. Therefore, the establishment of the milk detection model is of considerable significance to the identification of milk source and improvement of milk quality.

The traditional method to identify milk’s geographical origin is through physical tracking methods such as recording by experimenters. In recent years, many chemical analysis methods have been used to distinguish the origin of milk, such as the stable isotope ratio analysis method [10,11], the trace element content analysis method, and the nuclear magnetic resonance method [12]. At present, domestic and foreign research by near-infrared spectroscopy [13], microorganism physicochemical analysis [14,15], and DHI laboratory testing have achieved excellent results in quantitative detection of milk components [16]. However, these methods still have the disadvantages of high cost, low detection efficiency, vulnerability to damage, and cannot realize real-time detection of milk products. Therefore, it is essential to find a fast and efficient non-destructive testing method.

As a new gas detection and analysis technology, E-nose has reliable portability and simple operation, making food non-destructive testing easier [17,18,19]. The E-nose is a low cost digital electronic device that can mimic human olfaction. It can quickly evaluate complex, volatile gas mixtures and has been used in milk recognition, differentiation, and detection [20,21]. Bougrini et al. [22] used a hybrid E-nose and a voltammetric E-tongue to distinguish different pasteurized milk brands and their storage day. Tong et al. [23] analyzed the concentration of volatile substances in pre-heated skimmed milk using an E-nose and found that there was a good relationship between volatile compounds and sensory attributes through partial least squares regression (PLSR) model analysis. Although E-nose has been applied to the detection of dairy products, its performance still needs improvement. Analyzing E-nose signals using advanced machine learning techniques would enhance detection and estimation performance [24].

Therefore, this study proposes a fast identification method based on E-nose technology and machine learning techniques for milk source (dairy farm) identification and milk quality estimation. The developed E-nose system is mainly composed of a gas sensor array consisting of seven metal oxide semiconductor (MOS) sensors (FIGARO, Osaka, Japan) and a data acquisition module consisting of Arduino hardware and software modules. The collected gas information is transmitted to the PC through analog-to-digital conversion. After the data are preprocessed, pattern recognition algorithms are used for modeling to achieve the detection target. Based on three different classification algorithms: logic regression (LR), support vector machine (SVM), and random forest (RF), the milk source identification models are developed and compared. Gradient boosting decision tree (GBDT), extreme gradient boosting (XGBoost), and RF are used to construct models to estimate the content of milk fat and milk protein by utilizing historical data from E-nose measurements and DHI analytical measurements.

## 2. Materials and Methods

### 2.1. The Developed E-Nose

**Figure 1:**
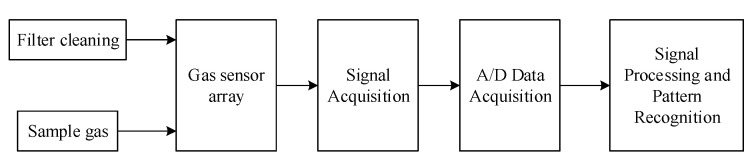
Structure diagram of E-nose system.

The developed E-nose is an electronic system that mimics the animal’s olfactory organs and uses sensor array responses to identify odors. The working process is as follows: firstly, the gas sensors of the sensitive element react with the sample gas, and then the response signal is transmitted to PC through analog-to-digital (A/D) converter, after data preprocessing, the model is built in combination with pattern recognition algorithm to complete detection. The E-nose developed (length: 30 cm, width: 20 cm, height: 20 cm) in this study is composed of a gas sensor array module, signal acquisition and data acquisition module, and signal processing and pattern recognition module, as shown in Figure 1.

The E-nose device designed in this study is divided into two layers. The upper layer is for gas collection, gas and sensor reaction, which includes transmission pipes, filter devices, intake pumps, exhaust pumps, and a gas chamber containing gas sensor arrays. Moreover, the upper wall is provided with two power ports and two air holes, respectively. The power ports are for providing power to the air pump. The air holes are divided into air intake holes and exhaust holes connected to the sampling test tube or the external environment. The lower layer is for the collection of response signals and data, including the Arduino development board and expansion board, and the USB connection port is set on the lower layer wall to be responsible for power supply and data transmission.

The sensitivity of each sensor in the array to the measured gas is different, so the system uses its response resistance value to identify the odor. In this study, the metal oxide semiconductor (MOS) sensors are selected as the E-nose gas sensors because of its advantages of fast response speed, high sensitivity, and strong stability. Ghasemi et al. [25] selected the E-nose device composed of TGS (Taguchi gas sensor) 2600, TGS2610, TGS2620 (FIGARO, Osaka, Japan) sensors to classify different types of cheeses. Sivalingam et al. [26] developed an E-nose prototype with an array of TGS 2620, 822, 813 (FIGARO, Osaka, Japan) sensors for real-time quality analysis of raw milk. Based on the characteristics of sensors and the above applications, seven MOS sensors (FIGARO, Osaka, Japan) were built into the E-nose gas sensor array. Table 1 shows the names of the gas sensors and the corresponding sensitive substances.

The sensitive element of the Figaro sensor is composed of SnO_2_ semiconductors. When the sample volatiles enter the collection system from the sampling tube and contact the heated metal oxide sensor array, the sensor resistivity G changes, and the ratio with the initial resistivity G_0_, G/G_0_ (relative resistivity) changes accordingly. When the gas concentration becomes larger, G/G_0_ deviates from 1 (greater than or less than 1). If the gas concentration is lower than the detection limit or there is no induction gas, it is close to or equal to 1.

The signal and data acquisition module uses Arduino hardware and software for design. In the developed E-nose system, the Arduino functions are: (1) obtaining the response values of the sensors; (2) processing data and communicating with the computer. The microcontroller on the development board is programmed using the Arduino programming language, compiled into a binary file, and passed to the microcontroller. Each sensor in the sensor array will digitally convert the response value to different volatile substances through a multiplexer analog-to-digital converter (ADC) and store the obtained data for subsequent computer analysis and identification and extraction of related features. The processed digital signal is transmitted to the upper computer through the serial port and finally presented on the serial port monitor.

The signal processing and pattern recognition module in the E-nose system plays a decision-making role. The original data from the sensors contain lots of complex information with high dimensions, and most of it is useless. Therefore, before inputting the data into the pattern recognition system, it is necessary to preprocess the original data, which mainly involves standardization processing, feature selection, feature dimensionality reduction, and retain valid information. The pattern recognition system can solve classification and regression problems by selecting different machine learning algorithms for modeling. According to different detection targets, setting reasonable model parameters can realize the binary classification problem and realize the multi-classification problem.

In addition, sample gas collecting and cleaning are realized by the gas collection system, which is composed of three parts: filter, air chamber, and air pump. Activated carbon is used as a gas desiccant to achieve gas filtration. The gas chamber is where the sample gas contacts and reacts with the sensor and with strong sealing performance to ensure gas concentration. The air pump provides power for gas transmission. The following parameter settings: cleaning time of 60 s, capture gas time of 90 s, and gas flow rate of 200 mL/min (range: 10 mL/min–1.1 L/min).

### 2.2. Milk Samples

Milk samples from 10 cattle farms’ raw milk in Hebei province were collected. The test cows to which the samples belong are lactating cows from 6 days postpartum to 6 days before milkless. The initial screening of these samples was carried out. Samples with low liquid levels and sub-standard temperatures were rejected. For DHI (Dairy Herd Improvement) machine detection, there will be a null value phenomenon, and the interference value needs to be eliminated before the experiment. Finally, 100 milk samples from each of 10 cattle farms were taken in the same period time for DHI analysis and E-nose measurement. In this study, three measurements for each milk sample were taken, and they were averaged to reduce measurement error. The DHI test samples and the E-nose test samples are the same.

#### 2.2.1. DHI Analytical Data

The milk composition feature (DHI analytical data) from the DHI laboratory analysis uses imported biochemical detection equipment, including milk composition analyzer, somatic cell counter, fresh-keeping cabinet, and other facilities. Milk sample test temperature is 40 ± 2 °C. It includes 6 test indicators: milk fat rate, protein rate, lactose rate, total solids, somatic cells (SCC), and urea nitrogen.

Milk fat contains linolenic acid, arachidonic acid, and various fat-soluble vitamins and phospholipids, which are needed by the human body [27]. The content of fat and protein is an essential indicator of evaluation for milk quality. In regular milk, the ratio of milk fat to milk protein is ranged from 1.12 to 1.30. If the value is too low, the cow may have rumen acidosis. The content of lactose in milk is generally between 4.6% and 5%. Its value not only affects milk production but also relates to rumen function. Cells are a collective term for macrophages, lymphocytes, and polymorphonuclear neutrophils in milk. The number of somatic cells (SCC) is an indicator of the extent of cow mastitis infection [28]. The number of SCC indicates the health status of cows’ milk, which is usually less than 50 × 10^4^/mL. Urea nitrogen of milk is derived from the blood, ranging from 10 mg/dL to 18 mg/dL [29]. Excessive urea nitrogen content proves that cows are more likely to suffer from acidosis [30].

#### 2.2.2. E-Nose Measurements

After heating in a 40 °C water bath, the sample gas is drawn. An amount of 20 mL of each milk sample was extracted and stored in a 40 mL test tube, sealed and placed for 10 min to ensure that the milk sample’s volatile matter filled the entire test tube. Before performing volatile gas capture, the airway and air chamber of the E-nose were cleaned with fresh air to eliminate interfering gas. 

**Figure 2:**
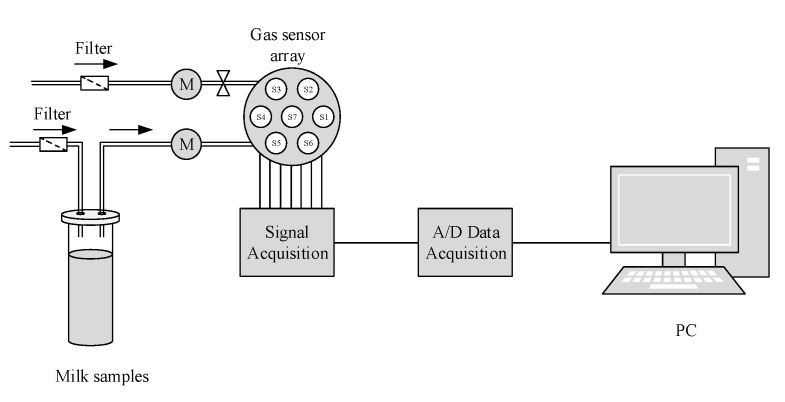
E-nose detection structure.

The measuring probe of E-nose and the balanced pressure tube were simultaneously extended into the headspace of the test tube. During the process of gas capturing, the filtered headspace gas of the milk sample is sucked into the gas chamber by the gas collection system, and contacts and reacts with the sensors. Then, the response value increases and tends to turn into a steady state, this process lasts for 90 s, and the gas flow rate is 200 mL/min. During the cleaning process, the filtered air gradually removes the volatile gas, and the response value decreases and stabilizes to a constant value, completing a sample measurement. This process lasts for 60 s. Three times for experiments were performed per sample, and the results averaged to reduce experimental errors. The E-nose detection process is shown in Figure 2. The obtained milk odor data are the relative resistivity ratios (G/G_0_) of the sensor array under the sample gas and the pure air environment in the steady state.

### 2.3. Data Analysis

In this study, 10 different sources of milk (dairy farms) were selected and the volatile gas in the milk samples was measured using an E-nose. A total of 1000 sample data after normalization (Equation (1)) were used for model development, of which 800 samples were used as the training set and the remaining 200 samples serve as the testing set.

(1) $$\frac{X-\bar{X}}{\sigma }$$

where *X* is the original data, $\bar{X}$ is the average of the original data, σ is the variance.

For the development of cattle farm classification models (or milk source identification models), principal component analysis (PCA) and linear discriminant analysis (LDA) are used to reduce the dimensions of model inputs. Three machine learning algorithms, LR, SVM, and RF, are then used to construct the classification models.

For the development of milk fat content and protein content estimation models using E-nose and DHI data, three nonlinear modeling algorithms, including GBDT, XGBoost, and RF, were used and compared.

#### 2.3.1. SVM

SVM is a supervised learning model that can perform pattern recognition, classification, and regression analysis [31,32]. The principle of SVM is to find the separation hyperplane that can correctly divide the classes in the training set and obtain the largest geometric distance. The objective function of the SVM is as follows:

(2) $$\begin{array}{c} \underset{w,b,\xi }{\min}\frac{1}{2}w^{T}w+C\sum_{i=1}^{n}\xi_{i}\\ s.t.y_{i}\left(w^{T}\phi \left(x_{i}\right)+b\right)\geq 1-\xi_{i},\xi_{i}\geq 0,i=1,2,\ldots ,n \end{array}$$

where *w* and *b* are the SVM parameters, $\xi_{i}$ is the classification loss of the *i*th sample point, $\phi \left(x_{i}\right)$ is the mapping function, *C* is the penalty parameter, *x* is the *i*th input sample, and *n* is the number of training samples.

For nonlinear classification problems, the kernel (mapping) function in SVM can map samples from the original space to high-dimensional space, making the samples linearly separable in the new space. Among them, the most commonly used and the most effective is the radial basis kernel function (RBF kernel):

(3) $$K\left(x_{1},x_{2}\right)=\exp\left(-\gamma {\left\|x_{1}-x_{2}\right\|}^{2}\right),\gamma >0$$

where $x_{1}$, $x_{2}$ are sample points of traing set; the parameter γ (gamma), defines the range of influence for a single training example, with low values meaning ‘far’ and high values meaning ‘close’.

#### 2.3.2. RF

Random forest is a crucial bagging-based ensemble learning method. It is composed of many decision trees (CARTs). It can be used to solve classification and regression problems and has strong anti-noise ability, can avoid overfitting. The procedure of developing an RF model is as follows: firstly, m sample points are extracted from the training sample set S to form a new training subset; secondly, a classification decision tree or regression model is constructed for each training subset, which is obtained by randomly selecting k features among all features as split nodes; the output of the model is the category (classification) with the highest number of votes or the average output (regression) of each decision tree [33].

#### 2.3.3. LR

Logistic regression is a supervised machine learning algorithm for solving classification problems. The principle is to find the minimum value of the loss function to make the prediction function more accurate, thereby solving the classification problem. The penalty term is a vital hyperparameter of the LR model, and the solver parameter can optimize the loss function [34].

#### 2.3.4. GBDT

Gradient boosting decision tree is an integrated boosting algorithm based on CART learner [35]. The purpose of its algorithm in each round of iteration is to minimize the loss function of the current learner so that the loss function always decreases along its gradient direction, and the final residuals approach 0 through continuous iteration, adding up all the tree results to get the final prediction.

#### 2.3.5. XGBoost

Extreme gradient boosting algorithm is an improved version based on GBDT, which is not sensitive to input requirements and is widely used in the industry. Compared with the general GBDT algorithm, XGBoost uses the second derivative of the loss function about the function to be sought, adds a regularisation term to prevent overfitting, and samples the attributes when constructing each tree. It has fast training speed and high accuracy and fitting effect, etc. [36].

## 3. Results and Discussion

### 3.1. Response Curve and Radar Chart Analysis of E-Nose

**Figure 3:**
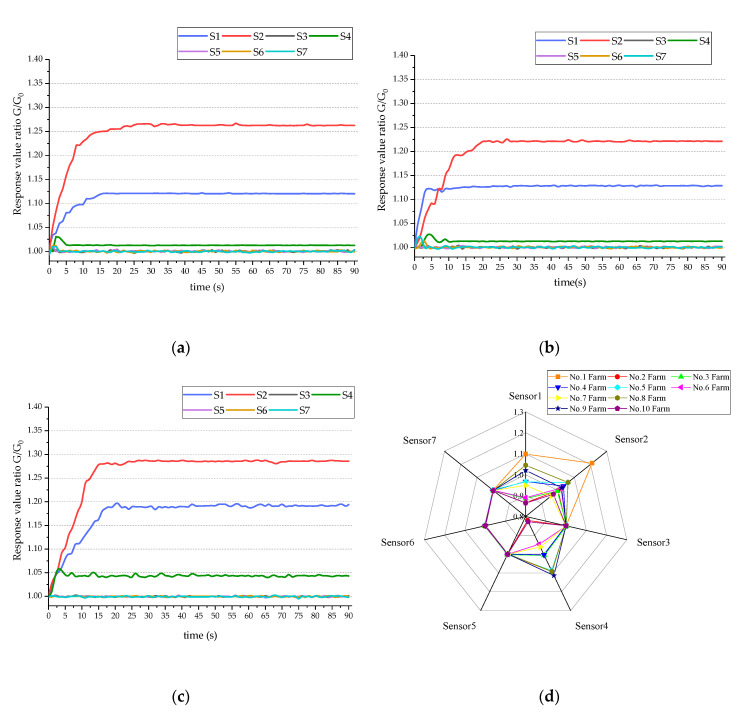
Response curve and radar chart for E-nose data: (**a**–**c**) response curve of E-nose; (**d**) radar chart of E-nose.

Figure 3a–c shows the sample’s response curves in 90 s sampling for three measurements. During the contact between the gas and the sensor surface, the ratio G/G_0_ (relative resistivity) keeps rising, and finally reaches a steady state in about 60 s. Among the seven sensors, the responses of S1, S2, and S4 are significant.

Steady-state values of E-nose sensor responses (collected at 90 s) for one sample randomly selected from each farm are used to produce a radar chart shown in Figure 3d, where each vertical axis represents a sensor. It can be seen from Figure 3d that the response values of sensor 1, sensor 2, and sensor 4 vary significantly with cattle farms. By observing the response curve and radar chart, the samples of different farms are distinguishable. Therefore, milk from different cattle farms could be identified based on E-nose measurement data.

### 3.2. Milk Source (Dairy Farm) Identification

In this study, the steady-state (90 s) value of the E-nose response is selected as the feature parameter of the E-nose and DHI analytical data as the composition of milk. A feature fusion method based on the milk component feature and odor feature is proposed to evaluate the identification of different cattle farms. During model construction, DHI analytical data, E-nose measurements, and fusion features are used as model inputs, respectively, to evaluate and compare model classification results. During data preprocessing, PCA and LDA are used to reduce the dimensions of the data for different features and retain valid information. Then milk source identification models are developed using support vector machine (SVM), random forest (RF), and logistic regression (LR) algorithms. The models are developed on the 800 samples of training data and tested on the remaining 200 samples of testing data to verify the developed models.

#### 3.2.1. Results of Data Dimensionality Reduction

The original DHI analytical data (six dimensions), the E-nose measurements (seven dimensions), and the DHI analytical and E-nose measurements fusion data (13 dimensions) were analyzed by PCA. The cumulative variance explanation rates of the first two principal components (PC) for these three cases are 99.908%, 95.96%, and 94.81%. Among them, PC1 and PC2 of DHI analytical data represent 99.9%, and 0.008% of the data variation respectively; PC1 and PC2 of E-nose measurements represent 88.38% and 7.58% of the data variation respectively; PC1 and PC2 of the fusion data represent 55.72% and 39.09% of the data variation respectively.

**Figure 4:**
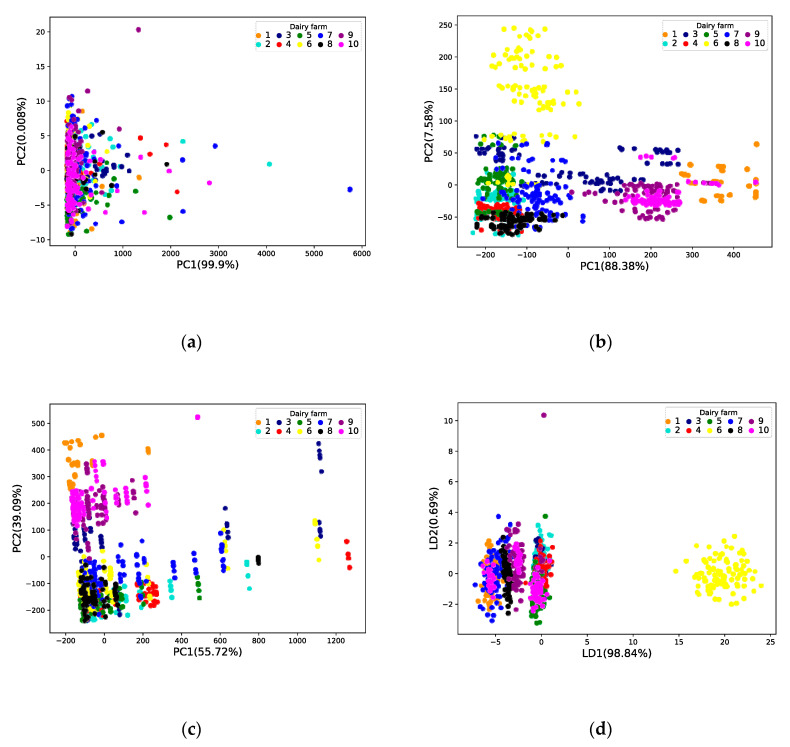

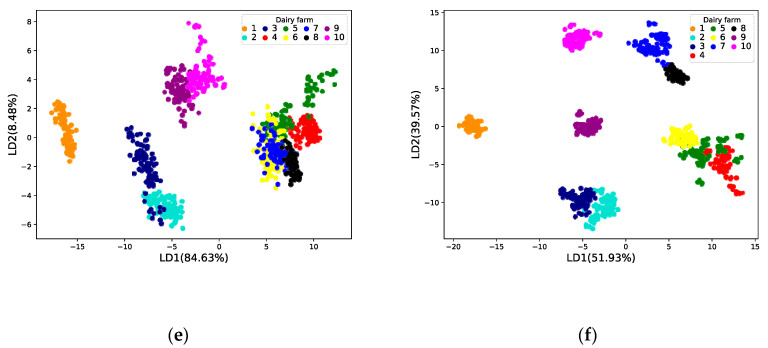
Visualization of data dimensionality reduction: (**a**) Daily Herd Improvement (DHI) data dimension reduction results by Principal Component Analysis (PCA); (**b**) E-nose data dimension reduction results by PCA; (**c**) Fusion data reduction results by PCA; (**d**) DHI data dimension reduction results by Linear Discriminant Analysis (LDA); (**e**) E-nose data dimension reduction results by LDA; (**f**) Fusion data reduction results by LDA.

Figure 4a–c shows the scatter plots in the principal component subspace, where the ten farms are color-coded. It can be seen from Figure 4a that the farms are randomly distributed and cannot be distinguished from the first two PCs of the DHI analytical data. Compared with Figure 4a, the first two PCs of the E-nose measurements in Figure 4b show more grouping of the farms, but it is still impossible to distinguish them. In Figure 4c, the first two PCs of the fusion data show more separations than the other two cases.

The LDA method was used to reduce the dimensionality of the original data, and the cumulative variance of the linear discriminant function in the three cases was 99.53%, 93.11%, and 91.5% (Figure 4d–f). In particular, LD1 and LD2 of DHI analytical data represent 98.84% and 0.69% of data variance respectively; LD1 and LD2 of E-nose measurements represent 84.63% and 8.48% of data variance respectively; LD1 and LD2 of the fusion data represent 51.93% and 39.57% of data variance respectively.

Although the original data after PCA dimensionality reduction is more comprehensive, the data distribution difference between different cattle farms after LDA dimensionality reduction is more significant. In particular, the dimensionality reduction results of the combined fusion data can achieve rapid differentiation, which proves that the samples are observed to be sufficiently representative, and the LDA dimensionality reduction method can be applied to milk sample data.

#### 3.2.2. Model Validation and Analysis

Each cattle farm draws 80 training sets and 20 testing sets, and a total of 800 training sets and 200 testing sets are available from the ten cattle farms. The SVM, RF, and LR methods are used to classify the milk sources after PCA and LDA dimension reduction. The classification accuracy is based on 200 testing set, and calculated as:

(4) $$Accuracy=\frac{TP}{TP+FP}$$

where *TP* (True Positive) is the number of times the dairy farm was correctly classified, *FP* (False Positive) is the number of times the dairy farm was incorrectly classified.

In the SVM classification model, radial basis function (RBF) is used as the kernel function of the model, the penalty parameter C and kernel parameter γ are set as 10 and 0.1 respectively, which give the best classification results.

The number of decision trees (N) is an important parameter of the RF-based model classification model. The larger N is, the better the model tends to perform. However, a high N value leads to longer training time and more memory consumption. It is found that the classification performance is best when the value of N is 4 in this study.

In the LR-based model, the parameters of the penalty term model are selected to meet L2 that meets the Gaussian distribution, avoid overfitting the model, and obtain results with more substantial generalization capabilities easily. Iteratively optimize the loss function by selecting a second-order derivative matrix.

The models of milk source identification are constructed with different features of milk, including odor features and composition features obtained from E-nose and DHI analysis, and fusion features, which are compared with the algorithm model based on these features after dimensionality reduction of PCA or LDA, including SVM, RF, and LR models, as shown in Table 2. During training, the five-fold cross-validation method is used to prevent overfitting. This method randomly divides the training set into five subsets, each time using different subsets as the validation set to obtain the accuracy rate, and finally acquires the mean of accuracy rate of each subset. The classification performance with PCA dimensionality reduction is significantly worse than that with LDA dimensionality reduction. The reason is that PCA does not consider the category during the dimensionality reduction process, and LDA is a supervised learning method with category output [37]. Each sample of the dataset for LDA has a category output. The LDA dimension reduction method is more geared towards classification than the PCA method.

When the input is the fusion features after LDA reduction, the model has the best classification performance with the accuracy of 95% for SVM, 94% for RF, and 92.5% for LR (based on the testing set). For the E-nose feature after LDA, the SVM model performs best with an accuracy rate of 85.75% for the training set and 85% for the testing set. For DHI feature after LDA, the accuracy ranged from 53.5% to 58.5% (based on the testing set), the training set and testing set have not achieved the classification effect. The results indicate that for milk source identification, SVM performs better than RF and LR models. The feature fusion method effectively solves the problem of missing information of a single feature. The fusion features contain both composition and odor information of milk, which effectively improves the classification effect of the model.

### 3.3. Estimation Models of Milk Fat Content and Protein Content by E-Nose

#### 3.3.1. Model Performance Indicators

In order to explore the established model, the following indicators are used to comprehensively evaluate the developed models.

(1) Mean Absolute Error (*MAE*) calculated as:
  (5) $$MAE=\frac{1}{n}\sum_{i=1}^{n}\left|y_{i}-{\hat{y}}_{i}\right|$$
(2) Mean Squared Error (*MSE*) calculated as:
  (6) $$MSE=\frac{1}{n}{\sum_{i=1}^{n}\left(y_{i}-{\hat{y}}_{i}\right)}^{2}$$
(3) Coefficient of Determination, *R*^2^ calculated as:
  (7) $$R^{2}=\frac{\sum_{i=1}^{n}{\left(y_{i}-{\hat{y}}_{i}\right)}^{2}}{\sum_{i=1}^{n}{\left(y_{i}-{\bar{y}}_{i}\right)}^{2}}$$

In the above equations, *n* is the number of samples, $y_{i}$ is the actual value, ${\hat{y}}_{i}$ is the predicted value, and ${\bar{y}}_{i}$ is the average of the actual value.

#### 3.3.2. Comparison of Different Models

**Figure 5:**
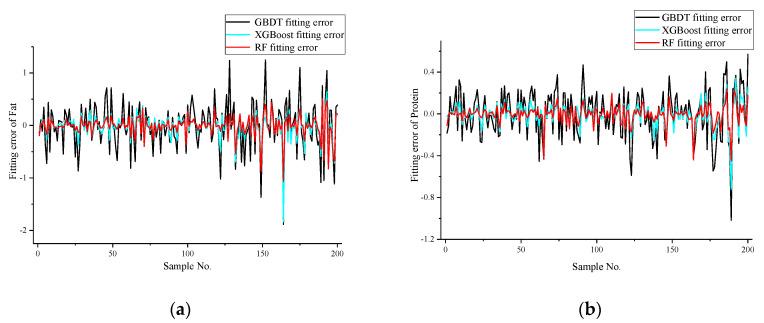
Model estimation error: (**a**) model errors for fat; (**b**) model errors for protein.

Based on the above evaluation indicators, the three models developed using GBDT, XGBoost, and RF are evaluated and compared. The milk fat content and protein content were used as the outputs of the models, and the seven sensor outputs from the E-nose were used as inputs to establish the milk quality estimation models. The performance indices of the developed models on the training set and testing set are shown in Table 3 and Table 4. The model errors on the testing set are shown in Figure 5.

As can be seen from Table 3 and Table 4, as well as Figure 5, among the different modeling methods, the RF method provides the best performance. Compared with GBDT and XGBoost models, the RF model has the smallest estimation error. Furthermore, it gives smaller *MAE*, *MSE* values, and larger R^2^ values than the other two modeling methods. In actuality, both the XGBoost and RF models estimate very well, with only a difference of 0.02 for milk fat and 0.01 for milk protein in R^2^ values. The results prove the effectiveness of the E-nose method to estimate the rate of milk fat and protein.

## 4. Conclusions

Milk source identification and estimation of milk fat content and protein content using an E-nose with machine learning techniques are studied in this paper. The E-nose developed is composed of a gas sensor array module, signal acquisition and data acquisition module, and signal processing and pattern recognition module. As for the rapid identification of milk source, LR, SVM, and RF are used, in conjunction with PCA and LDA dimension reduction, to construct the classification models. Classification models using DHI features, E-nose features, and fusion features are investigated and compared. It is shown that milk source identification models using LDA extracted features as inputs give better performance than those using PCA extracted features as inputs. The reason is that, in contrast to PCA, LDA is a supervised learning method considering the categories of the data samples. The results show that the SVM model based on the fusion features after LDA has the best performance with an accuracy of 95%. Therefore, the feature fusion method can effectively improve the classification effect of the model.

For the estimation of milk fat content and protein content using E-nose data measurement, GBDT, XGBoost, and RF algorithms were used to establish the estimation models. The RF model has the best fitting performance with the R^2^ values being 0.9399 and 0.9301 for fat and protein content, respectively. The experimental results show that milk quality can be accurately estimated from E-nose measurements using machine learning techniques. Further works on enhancing model accuracy and reliability will be carried out.

## Figures and Tables

**Table 1:** Gas sensor information in E-nose system.

No.	Sensor	Sensitive Substance
1	TGS2600	Polluting gas
2	TGS822	Volatile substances of alcohol and organic solvents
3	TGS2611	Methane gas
4	TGS826	Ammonia
5	TGS2602	Volatile organic compounds (VOC), benzene
6	TGS832	Freon gas
7	TGS2620	Alcohol, carbon monoxide, other volatile organic vapors

**Table 2:** Accuracy (mean of five-fold cross-validation) in milk source identification based on PCA and LDA (%).

Features		SVM		RF		LR	
		Train	Test	Train	Test	Train	Test
DHI	PCA	19.50	15.50	17.63	18.50	19.88	18.00
	LDA	57.75	58.50	52.13	53.50	53.38	56.00
E-nose	PCA	56.25	59.50	71.62	70.50	62.00	65.00
	LDA	85.75	85.00	82.13	80.50	84.38	81.50
Fusion	PCA	41.50	45.00	53.38	51.50	39.75	34.50
	LDA	95.50	95.00	92.50	94.00	93.50	92.50

**Table 3:** Estimation models for fat content based on three algorithms.

Model	Training Set			Testing Set		
	*MAE*	*MSE*	*R* ^2^	*MAE*	*MSE*	*R* ^2^
GBDT	0.3267	0.1907	0.7201	0.3245	0.1926	0.7172
XGBoost	0.1063	0.0241	0.9645	0.1487	0.0573	0.9158
RF	0.1046	0.0253	0.9627	0.1253	0.0410	0.9399

**Table 4:** Estimation models for protein content based on three algorithms.

Model	Training Set			Testing Set		
	*MAE*	*MSE*	*R* ^2^	*MAE*	*MSE*	*R* ^2^
GBDT	0.1773	0.0498	0.7003	0.1770	0.0501	0.6985
XGBoost	0.0616	0.0071	0.9572	0.0766	0.0123	0.9257
RF	0.0488	0.0052	0.9687	0.0607	0.0116	0.9301
